# Practical pH Testing for Nasogastric Tube Verification: A Prospective Method‐Comparison Study of Low‐Cost Handheld Meters and Colourimetric Strips

**DOI:** 10.1002/hsr2.72215

**Published:** 2026-03-27

**Authors:** Luca D. Zambetti

**Affiliations:** ^1^ Department of Internal Medicine Frere Hospital East London South Africa; ^2^ Department of Internal Medicine Cecilia Makiwane Hospital East London South Africa; ^3^ Department of Internal Medicine and Pharmacology Walter Sisulu University East London South Africa

**Keywords:** gastric aspirate, nasogastric tube, patient safety, pH measurement, pH meter, pH test strip, tube verification

## Abstract

**Background and Aims:**

Nasogastric (NG) tubes are widely used for enteral nutrition, medication administration, and gastric decompression, but misplacement can cause serious, preventable harm. International guidelines recommend gastric aspirate pH testing with pH strips to reduce reliance on X‐rays, but subjective color interpretation and stock shortages limit their reliability in resource‐constrained settings. Commercial handheld pH meters offer objective digital readings and reusability, but their performance in routine clinical practice is uncertain.

**Methods:**

A prospective method‐comparison study was conducted among adult inpatients requiring NG tube insertion in a South African public hospital. Gastric aspirate pH was measured using pH strips, followed immediately by a commercially available handheld pH meter on the same sample. Agreement was assessed using Bland–Altman analysis and Cohen's kappa, and diagnostic accuracy at a threshold of pH ≤ 5.5 was secondarily explored using routinely obtained chest radiography, where available, as the reference standard.

**Results:**

Forty‐five patients were enrolled; gastric aspirate was obtained from 40 (89%), and 26 (58%) had both an aspirate and an interpretable chest X‐ray. The handheld pH meter showed a mean bias of +0.17 pH units, with 95% limits of agreement from –0.46 to +0.80. At pH ≤ 5.5, categorical agreement with pH strips was substantial (κ = 0.754; *p* < 0.001). Diagnostic accuracy estimates for the pH meter when using radiology as the standard were limited by the small radiograph sample, but showed a sensitivity of 80.0% (95% CI 56.3–94.3%), a specificity of 100% (54.1–100%), a positive predictive value of 100% (79.4–100%) and a negative predictive value of 60.0% (26.2–87.8%). pH strips performed similarly.

**Conclusions:**

In this cohort, a low‐cost handheld pH meter showed a small mean bias and substantial categorical agreement with pH strips at pH ≤ 5.5. Its objective digital readout and reusability address key limitations of color‐based strips and support its use in resource‐constrained settings as a practical bedside tool for confirming gastric NG tube placement. However, pH values > 5.5 do not reliably exclude gastric placement, and chest radiography remains necessary when results are inconclusive or discordant with clinical expectations.

**Abbreviations**
CI: confidence interval
IQR: interquartile range
LoA: limits of agreement
NG: nasogastric
NPV: negative predictive value
PPV: positive predictive value
SD: standard deviation

## 1 Introduction

Nasogastric (NG) tubes are single‐lumen plastic tubes inserted through the nose and passed via the pharynx and esophagus into the stomach. They are widely used in healthcare for enteral feeding, medication administration, and gastric decompression [11, 18]. Correct verification of tube tip position is essential because malposition can cause serious, preventable harm [19]. Serious complications most often occur when NG tubes are inadvertently placed in the respiratory tract, leading to bronchial aspiration, pneumothorax, or pleural effusion [13].

The importance of accurate NG tube placement has led to the development of multiple methods to confirm tip position. Methods to verify appropriate NG tube placement need to be reliable and to perform consistently without logistical complexity or dependence on sophisticated equipment or laboratory infrastructure [12]. A properly obtained and interpreted X‐ray is widely regarded as the gold standard as it allows direct visualization of the NG tube tip [11, 18]. However, routine radiographic confirmation for every insertion is often impractical in resource‐limited health systems due to delayed access, increased workload, and additional costs [8]. For this reason, international guidelines recommend the cost‐aware strategy of bedside testing of gastric aspirate pH as an initial verification step, with radiography reserved for inconclusive results or high‐risk scenarios where gastric acidity may be altered [11].

Colourimetric pH strips are commonly used for aspirate testing, but two limitations reduce their reliability in real‐world practice. First, interpretation depends on subjective color matching, making them vulnerable to inter‐ and intra‐observer variability and clinically important misinterpretation. This issue has been demonstrated consistently across method‐comparison studies spanning more than four decades [2, 3, 4, 5, 9, 15, 16, 17]. Second, pH strips are single‐use consumables, and consistent availability of appropriate strips cannot be assumed in many public‐sector settings, where stock shortages are common.

Commercially available handheld pH meters are inexpensive battery‐operated devices that are reusable, easy to calibrate, and provide an objective readout. These features potentially address both interpretation error and consumable supply vulnerability. Previous studies have shown that handheld pH meters provide closer agreement with laboratory reference pH measurements than colourimetric strips, but the devices evaluated to date have been expensive laboratory‐grade instruments that are not designed for routine bedside use [4, 5, 17].

This study aimed to address this knowledge gap by evaluating agreement between a low‐cost commercial handheld pH meter and standard colourimetric pH strips for gastric aspirate measurement during NG tube position verification. Diagnostic accuracy against chest radiography was included as a secondary, exploratory analysis to contextualize clinical classification performance rather than to re‐establish the validity of pH‐based verification, which is already supported by existing evidence. The study was undertaken in a South African public hospital where shortages of appropriate pH strips underscored the need for pragmatic alternatives. By assessing an affordable, reusable device in a resource‐limited setting, this study contributes evidence relevant to improving the quality and safety of a widely used clinical procedure.

## 2 Methods

### 2.1 Study Design

This was a single‐center, prospective method‐comparison study evaluating the agreement between a low‐cost handheld pH meter and standard colourimetric pH strips for gastric aspirate pH measurement during NG tube placement. When available, a chest X‐ray was used as the reference standard to determine the NG tube tip position. This study was designed and reported in accordance with the STARD 2015 reporting guideline [6]. Ethics approval was obtained from a university research ethics committee.

### 2.2 Setting

The study was conducted from October 2025 to November 2025 in a 900‐bed public hospital in South Africa. The hospital serves a predominantly low‐income urban and peri‐urban population and functions as a tertiary referral center for surrounding primary care clinics and district hospitals. It is typical of resource‐limited public‐sector facilities, with constrained access to timely radiographic imaging and variable availability of disposable diagnostic consumables.

### 2.3 Participants and Eligibility Criteria

Consecutive adult medical inpatients (aged ≥ 18 years) who required NG tube insertion as part of routine clinical care were eligible for inclusion. Written informed consent was obtained from all participants or their legally authorized representatives prior to enrollment.

Patients in whom NG tube placement was considered unsafe or contraindicated were excluded. Patients who were moribund, lacked a suitable surrogate decision‐maker, or for whom informed consent could not be obtained were also excluded. No NG tubes were inserted solely for the purposes of the study.

### 2.4 Sample Size

The target sample size of 45 patients was informed by methodological guidance for Bland–Altman agreement analysis [1]. Previous comparisons of laboratory handheld meters and pH strips reported mean differences of 0.17–0.50 pH units and standard deviations (SDs) of 0.69–0.91 [4, 15, 17]. Using a conservative SD of 0.9 and an expected mean difference of 0.3, and assuming 95% confidence and 80% power, approximately 36 paired measurements were required to estimate the mean bias and 95% limits of agreement with acceptable precision.

A parallel calculation was performed for the categorical endpoint (pH ≤ 5.5), using Cohen's kappa. Assuming an expected kappa of 0.6, a null hypothesis kappa of 0.4, and an agreement proportion of 0.75, the required sample size was approximately 37 participants. To account for anticipated data loss—particularly in patients from whom gastric aspirate could not be obtained—the sample size was increased by 20%, yielding a final target of 45 participants.

### 2.5 pH Measurement Instruments

**Figure 1:**
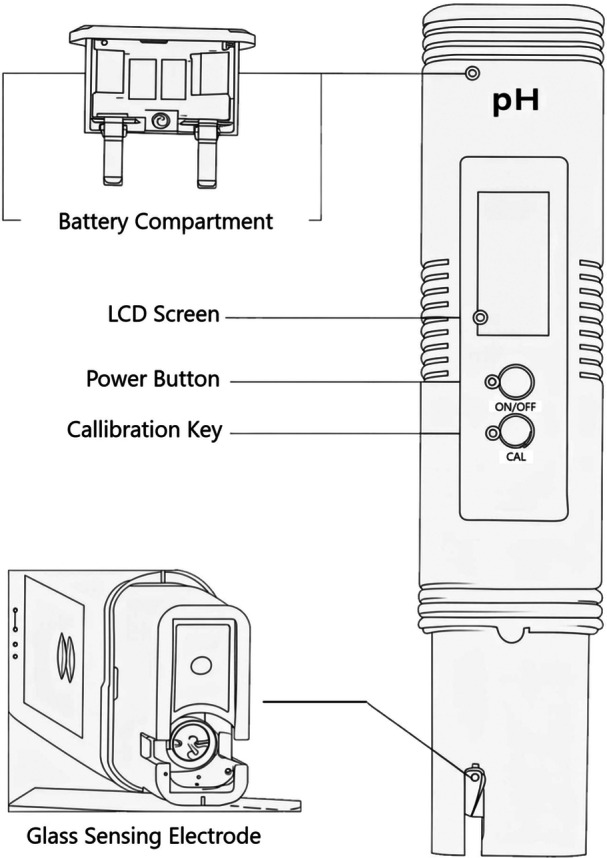
Schematic of the handheld digital pH meter used for gastric aspirate pH measurement. The diagram depicts the meter body, LCD screen, power button, calibration key, battery compartment, and glass sensing electrode. Author‐created schematic.

The instruments used for data collection included a generic colourimetric pH strip set (pH range 0–14 in 0.5‐unit increments) and a commercial handheld digital pH meter (Figure 1). Both were procured from South African online retailers in September 2025 at a cost of R212 for a box of 100 strips and R190 for the meter (including buffer solutions).

Before patient recruitment, a simple pre‐study bench validation of both pH measurement methods was undertaken using standard buffer solutions (target pH 4.00, 6.86, and 9.18). This was to confirm that both devices performed within acceptable analytic limits before clinical testing. The meter was tested after rinsing with distilled water and again after rinsing with tap water to reflect likely real‐world use. Full validation results are provided in *Appendix A*.

The handheld pH meter was calibrated before participant enrollment and recalibrated on three occasions (approximately weekly) during the data‐collection period, using standard buffer solutions in accordance with the manufacturer's instructions. The timing of recalibrations was pragmatic and usage‐based rather than protocolised. No suspected drift or instability was noted during bedside use over the study period. The manufacturer recommends recalibration after prolonged inactivity, very frequent use, when high accuracy is required, or if inadvertent calibration is triggered (e.g., by pressing the calibration button while the electrode is exposed to air).

### 2.6 Nasogastric Tube Insertion and Clinical Procedures

Potential participants were identified by the usual care teams, who inserted, secured, and managed NG tubes according to local practice. The hospital did not utilize a standardized NG tube confirmation protocol during the study period, and chest radiography was not mandated by the study to avoid unnecessary radiation exposure. Because imaging was requested at the clinician's discretion, the subgroup undergoing chest radiography may not represent the full cohort. Diagnostic accuracy estimates derived from this subgroup are therefore susceptible to partial verification bias.

Following tube insertion, the primary investigator attempted to obtain a gastric aspirate using a syringe attached to the NG tube. When aspirate was not obtained on the first attempt, standard manoeuvres—such as repositioning, air insufflation, and reattempting using slow negative pressure—were performed to improve yield. Patients from whom no aspirate could be obtained for pH measurement were recorded but excluded from the primary agreement analyses.

### 2.7 Bedside pH Measurement (Index Tests)

Index tests were performed immediately after obtaining the gastric aspirate. A pH strip was briefly (approximately half a second) soaked in the collected fluid, and the color was matched to the manufacturer's scale to obtain a pH value. The pH meter electrode was then immersed in the fluid, and the stabilized digital reading was recorded. This sequence was chosen to minimize bias from digital readings influencing subjective strip interpretation, although a bias towards greater accuracy for the strips was likely introduced by a single interpreter. The investigator performing the index tests was blinded to chest X‐ray results at the time of pH measurement.

### 2.8 Chest X‐Ray (Reference Standard)

For participants who underwent chest radiography as part of routine care, images were reviewed by the primary investigator using standard radiological criteria; expert radiology services were not available at the hospital. The investigator was not blinded to clinical information or pH results. An NG tube was classified as gastric if it followed the expected esophageal course, crossed the diaphragm at the midline, and terminated below the left hemidiaphragm [10].

Any suspected malpositions were promptly communicated to the clinical team, with subsequent management left to their discretion. Because chest radiographs were obtained for clinical rather than study purposes, the timing of imaging in relation to pH testing was neither standardized nor recorded. In routine practice at the study site, imaging is typically requested shortly after insertion and before initiation of feeding; however, variability in timing may have occurred. Patients without interpretable X‐rays were documented but excluded from the diagnostic accuracy analyses.

### 2.9 Outcomes

The primary outcome was agreement between gastric aspirate pH measurements obtained using the handheld pH meter and those obtained using colourimetric pH strips.

Secondary outcomes included (i) categorical agreement between methods at the guideline‐recommended threshold of pH ≤ 5.5 and (ii) exploratory diagnostic accuracy metrics for each method against chest radiography. The radiographic comparison was included to contextualize threshold‐based classification performance rather than to establish definitive diagnostic properties.

### 2.10 Statistical Analysis

Data were captured in Microsoft Excel and analyzed using IBM SPSS Statistics for Windows, version 31.0 (IBM Corp., Armonk, NY, USA). Continuous variables were summarized using means and SDs or medians and interquartile ranges (IQRs), as appropriate, and categorical variables as frequencies and percentages.

Agreement between pH measurements obtained by the handheld meter and pH strips was assessed using Bland–Altman analysis [1]. The mean difference (bias) and 95% limits of agreement (LoA), calculated as mean difference ±1.96 SDs, were reported. LoA within ±0.5 pH units were considered clinically acceptable, as differences of this magnitude would not typically influence classification around the pH ≤ 5.5 threshold used for NG tube verification [11, 14].

For the threshold analysis, each pH value was categorized as ≤ 5.5 or > 5.5. Categorical agreement between methods was evaluated using Cohen's kappa with 95% CIs. For participants with interpretable chest radiographs, 2×2 contingency tables were constructed for each method, and sensitivity, specificity, PPV, and NPV with exact (Clopper–Pearson) 95% CIs were calculated using chest X‐ray classification (gastric vs non‐gastric placement) as the reference standard. All statistical tests were two‐sided, with significance set at *p* < 0.05.

All analyses, including Bland–Altman agreement and diagnostic accuracy calculations, were pre‐specified.

## 3 Results

### 3.1 Participant Enrollment and Flow

**Figure 2:**
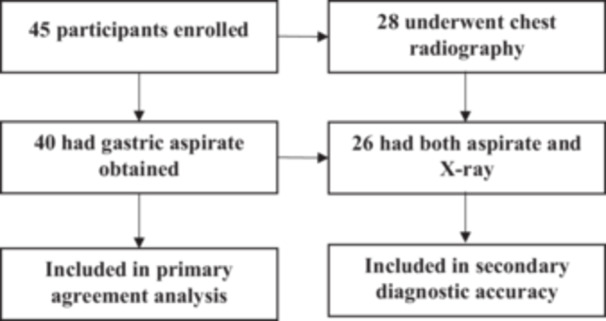
Study flow diagram for enrollment and inclusion in analyses.

Forty‐five adult medical inpatients were enrolled (Figure 2). Gastric aspirate was successfully obtained from 40 participants (88.9%), generating 40 paired pH measurements for analysis. Chest radiography was performed in 28 (62.2%) patients as part of routine care; 26 of these had both an obtainable gastric aspirate and interpretable X‐ray and were included in the diagnostic accuracy analyses.

### 3.2 Participant Characteristics and Gastric pH Measurements

The mean age of the enrolled participants was 51 years (SD 21.3; range 18–93), and 25 (56%) were male. The most common primary admission diagnoses were stroke (27%) and delirium (11%), with the remainder comprising infectious, neurological, and toxic‐metabolic conditions. The predominant indication for NG tube insertion was altered consciousness (93%), followed by dysphagia (4%) and gastric decompression (2%).

Gastric aspirate was not obtained for 5 participants, precluding pH measurement. Among the 40 participants with paired pH measurements, all strip‐based and meter‐based readings were numeric with no indeterminate results. The distribution of pH values for both methods is summarized in Table 1, demonstrating comparable overall pH ranges and variability, with similar central tendencies across a wide but clinically expected range of gastric acidity. Among the 28 patients who underwent chest radiography, 21 had gastric placement, and 7 had confirmed malposition (esophageal or duodenal).

**Table 1:** Summary of age and gastric aspirate pH measurements for enrolled participants.

Variable	*N*	Minimum	Maximum	Mean	SD
**Age (years)**	45	18	93	51.13	21.33
**pH (pH strip)**	40	1.00	7.00	4.79	1.81
**pH (handheld pH meter)**	40	1.07	7.14	4.96	1.89

Abbreviations: SD, standard deviation; *N*, number of observations.

### 3.3 Agreement between Methods

**Figure 3:**
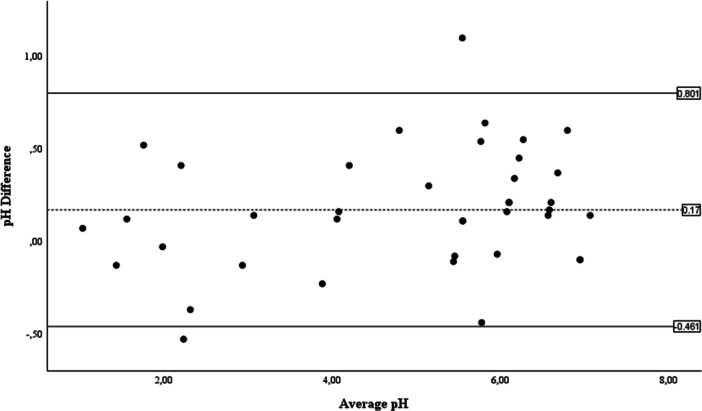
Bland–Altman plot of the agreement between the handheld pH meter and pH strips. Bland–Altman plot showing the difference between handheld pH meter and pH strip pH measurements (meter minus strip) plotted against the mean of the two methods for gastric aspirate samples (*n* = 40). The dashed line indicates the mean difference (bias), and the solid horizontal lines indicate the 95% LoA (–0.46 to +0.80 pH units).

Bland–Altman analysis (Figure 3) demonstrated a modest mean difference of +0.17 pH units (95% CI 0.07–0.27), indicating minimal systematic bias. The 95% LoA ranged from –0.46 (95% CI –0.64 to –0.28) to +0.80 (95% CI + 0.62 to +0.98) pH units. While the lower limit fell within the prespecified ±0.5 pH‐unit margin for clinical acceptability, the upper limit exceeded this threshold, indicating that at the extremes of variability, differences between methods could approach 0.8 pH units. However, when evaluated at the clinically relevant decision threshold of pH ≤ 5.5, categorical agreement between methods remained substantial (κ = 0.754; 95% CI 0.56–0.95; *p* < 0.001).

### 3.4 Diagnostic Accuracy in Participants with Chest X‐Rays

Of the 26 participants with paired pH measurements and interpretable chest X‐rays, the prevalence of gastric placement was 20 (77%). Both pH strips and the handheld meter demonstrated excellent specificity and PPV, with no false‐positive gastric classifications against the reference standard. Sensitivity was moderate for both methods, with the strips performing slightly better than the meter. No cases of false reassurance of gastric placement occurred in this subset, and no adverse events related to pH testing were observed. Full diagnostic accuracy metrics are shown in Table 2.

**Table 2:** Diagnostic accuracy of the handheld pH meter and pH strips at a threshold pH ≤ 5.5 using chest X‐ray as the reference standard (*n* = 26).

Measure	pH Meter	95% CI	pH Strips	95% CI
**Sensitivity**	80.0%	56.3–94.3%	85.0%	62.1–96.8%
**Specificity**	100%	54.1–100%	100%	54.1–100%
**PPV**	100%	79.4–100%	100%	80.5–100%
**NPV**	60.0%	26.2–87.8%	66.7%	29.9–92.5%

Abbreviations: CI, confidence interval; NPV, negative predictive value; PPV, positive predictive value.

## 4 Discussion

### 4.1 Key Findings

This study evaluated a low‐cost handheld pH meter against standard colourimetric pH strips for measuring the pH of gastric aspirate during NG tube placement in a South African public hospital. In 40 patients with paired measurements, the mean difference between methods was small (+0.17 pH units), indicating that the handheld meter tended to read slightly higher than the strips. Although the upper LoA exceeded the prespecified ±0.5 pH‐unit margin, the mean bias was small. The ±0.5 margin was intentionally conservative, reflecting the 0.5‐unit resolution of colourimetric strips and the proximity of the 5.5 threshold used for clinical classification. Importantly, substantial categorical agreement at this threshold indicates that, in practice, the observed variability rarely resulted in discordant classification. These findings suggest that while continuous numeric equivalence between methods was not perfect, agreement was adequate for threshold‐based clinical decision‐making.

**Figure 4:**
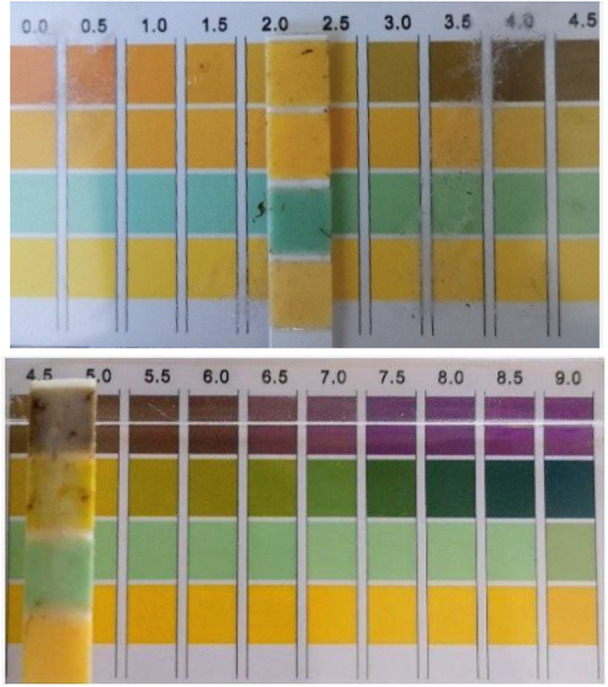
Photographic examples of pH strip color interpretation.

The discrepancies between readings were most apparent in samples with heterogeneously pigmented gastric aspirate, where strip interpretation was particularly challenging. The observed dispersion in differences is consistent with a known practical limitation of colourimetric strips: subjective color matching to the manufacturer's color scale can be difficult in heterogeneously pigmented aspirates. Figure 4 illustrates this challenge and underscores the practical limitations of relying on subjective color matching.

While this study did not include a laboratory reference standard to adjudicate which method was closer to “true” pH in discrepant cases, the pattern of disagreement observed at the bedside—together with existing evidence of inter‐observer variability for strips—suggests that some of the variability may plausibly arise from strip interpretation rather than meter instability. This supports the rationale for using an objective digital readout to reduce interpretation‐related error in routine practice.

Categorical agreement between methods at the guideline‐recommended threshold of pH ≤ 5.5 was substantial (κ = 0.754). The handheld pH meter demonstrated excellent specificity (100%) and PPV (100%), indicating that low pH readings can reliably confirm gastric placement. Sensitivity (80.0%) and NPV (60.0%) were more modest, so readings above 5.5 should not be relied on to exclude gastric positioning. The pH strips showed very similar performance, with slightly higher sensitivity and NPV, suggesting that the reduced sensitivity at higher pH values is an inherent limitation of pH‐based confirmation rather than a defect of the handheld meter.

Importantly, this study was not designed to re‐validate pH testing as a diagnostic strategy for NG tube confirmation, as this has been extensively evaluated and is incorporated into international guidelines [11]. Rather, the purpose was to determine whether a low‐cost handheld pH meter performs sufficiently similarly to colourimetric strips to serve as a practical substitute. The X‐ray comparison was therefore intended to ensure that substitution would not introduce clinically significant misclassification, rather than to derive definitive estimates of sensitivity or specificity.

### 4.2 Comparison with Previous Research

The wider‐than‐ideal limits of agreement observed in this study are consistent with earlier work showing substantial variability in gastric aspirate pH under real‐world conditions, where admixture with feed, bile, blood, and medications contributes to greater dispersion at higher pH values [2, 5, 16]. The small positive bias of the handheld meter aligns with previous studies in which electrode‐based measurements tended to yield slightly higher pH values than colourimetric methods, particularly in dilute or heterogeneous samples [4, 16, 17].

The threshold of pH ≤ 5.5 used in this study corresponds to the cut‐off recommended in international guidelines for identifying gastric NG tube placement [11, 14]. This threshold is intentionally conservative, designed to improve specificity and reduce the risk of catastrophic misfeeding into the respiratory tract, rather than to exclude gastric placement when values are higher. The high specificity and PPV observed in this study mirror previous findings demonstrating that low pH values reliably confirm gastric placement, whereas more modest sensitivity and NPV reflect the well‐described limitation that some genuinely gastric aspirates register above 5.5 on both strips and meters [5, 16, 17].

Earlier comparative studies evaluated laboratory‐grade pH meters, which, although likely more accurate, are expensive and impractical for widespread bedside use [2, 7, 15]. This study extends the literature by showing that a low‐cost, commercially available handheld meter can achieve clinically useful agreement with colourimetric strips, addressing a practical need for accessible and reliable NG tube confirmation tools in resource‐limited settings.

### 4.3 Strengths and Limitations

This study was conducted in a real‐world, resource‐constrained public hospital, enhancing relevance to routine clinical practice. The cohort largely reflects internal medicine inpatients requiring short‐term enteral access. While differing pathophysiology may alter gastric pH distributions (and therefore the sensitivity of pH‐based confirmation) in other populations, agreement between measurement methods is unlikely to depend strongly on indication.

Methodologically, the use of paired measurements from the same gastric aspirate eliminated between‐sample variability, and the application of complementary statistical approaches (Bland–Altman analysis and Cohen's kappa) provided a robust assessment of agreement between measurement methods.

Several limitations must be acknowledged. The sample size was modest, and chest radiography was performed in only 28 of 45 participants as part of routine clinical care. Because imaging was requested selectively rather than protocolised for all participants, the diagnostic accuracy analysis is subject to partial verification bias. Clinicians may have been more likely to request radiography when aspirate could not be obtained, when pH values exceeded the 5.5 threshold, or when clinical suspicion of malposition was high. As a result, the imaged subgroup may not represent the full cohort and may have been enriched by diagnostically challenging cases. Accordingly, the radiographic diagnostic accuracy findings should be interpreted as exploratory and hypothesis‐generating. Importantly, the primary method‐comparison agreement analysis was not affected by verification bias, as paired pH measurements were obtained from the same aspirate in all included cases.

Furthermore, the absence of standardized or recorded timing between bedside pH measurement and chest radiography introduces potential temporal misclassification. Tube migration or changes in gastric acidity between the two assessments could theoretically result in discordant classification unrelated to intrinsic test performance. Although imaging at the study site is generally performed soon after insertion and before feeding initiation, variability in timing cannot be excluded.

Finally, only one handheld pH meter model and one brand of colourimetric strips were evaluated, and all measurements and X‐ray interpretations were performed by a single investigator at a single center. This precludes assessment of inter‐rater reliability and introduces potential observer bias, despite the predefined measurement sequence and blinding of pH measurements to radiographic findings at the time of testing. Although the study setting is representative of many resource‐limited public hospitals, generalizability to other health systems and device models should be made with caution.

### 4.4 Implications for Primary Care and Hospital Services

Overall, the findings of this study support the ongoing use of pH‐based confirmation as a guideline‐concordant rule‐in strategy for verifying gastric placement. A low‐cost handheld pH meter, which provides an objective digital reading and is reusable, can reliably fulfill this role while directly addressing two major limitations of colourimetric strips: inconsistent availability and subjective color interpretation. However, because sensitivity remains moderate, chest radiography is still required when pH readings are inconclusive or discordant with clinical expectations, and continues to represent the gold standard for confirming NG tube tip position.

**Figure 5:**
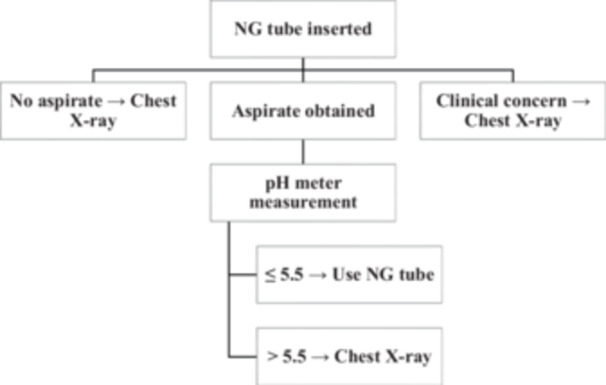
Proposed integration of a handheld pH meter into nasogastric tube verification pathways.

To enhance practical applicability, Figure 5 illustrates a proposed integration pathway in which a handheld pH meter functions as a rule‐in tool within a standardized nasogastric tube verification algorithm. In this approach, successful aspiration of gastric content with a pH ≤ 5.5 permits use of the tube, whereas values > 5.5 or failure to obtain an aspirate prompt radiographic confirmation. This reflects existing guideline‐based practice while substituting an objective digital reading for subjective color interpretation.

Electrode drift is a recognized limitation of reusable pH meters, although calibration checks performed before and during this study did not suggest instability. For routine implementation, a pragmatic approach would be recalibration approximately every 2 weeks (adjusted to usage volume), and additionally whenever readings appear unstable or after prolonged inactivity, consistent with manufacturer guidance.

Although a formal cost‐effectiveness analysis was not conducted, a simple cost comparison provides relevant context. At the time of procurement, 100 colourimetric pH strips cost R212 (approximately US$11; ~US$0.11 per test), whereas the handheld pH meter cost R190 (approximately US$10) as a one‐off purchase, with calibration solutions included (exchange rate approximately R19/US$ at time of purchase). On this basis, the device reaches cost parity after approximately 90 uses, after which the marginal per‐test cost becomes negligible. In moderate‐ or high‐volume settings, this may translate into meaningful cost savings over time. In addition, reliance on a reusable device reduces vulnerability to consumable stock shortages, a recurring challenge in resource‐limited health systems. However, device lifespan and long‐term maintenance costs were not formally evaluated and warrant further study.

Given the widespread availability and affordability of commercial handheld meters, these findings may help re‐establish or strengthen pH‐based NG tube confirmation pathways in other resource‐constrained health systems where strip supply is unreliable, thereby supporting safer and more standardized care. They also provide a practical evidence base to inform the development of high‐quality, context‐appropriate guidelines for NG tube placement in low‐resource settings.

### 4.5 Future Research

Future research should evaluate multiple low‐cost meter models, assess usability and inter‐operator reliability, and investigate cost‐effectiveness compared with existing confirmation methods. Larger, multi‐center studies with protocolised imaging would allow more precise estimation of diagnostic accuracy and better characterization of how handheld meters can be integrated into NG tube verification algorithms in resource‐limited health systems.

## 5 Conclusion

A low‐cost handheld pH meter demonstrated a modest mean bias and substantial categorical agreement with colourimetric pH strips and can be used as a practical rule‐in tool for confirming gastric placement when the pH is ≤ 5.5. Its objective digital readout, reusability, and widespread commercial availability offer clear practical advantages over colourimetric strips, particularly in settings where strip supply is unreliable or color interpretation is challenging. However, readings above pH 5.5 cannot reliably exclude gastric placement, underscoring the continued need for chest radiography when measurements are inconclusive. Overall, this study supports the integration of a low‐cost handheld pH meter as a practical and accessible alternative to pH strips within NG tube confirmation pathways, especially in resource‐limited settings.

## Author Contributions

**Luca D Zambetti:** conceptualization, investigation, writing – original draft, methodology, writing – review and editing, validation, formal analysis, resources, data curation, visualization, project administration.

## Funding

The author received no specific funding for this work.

## Ethics Statement

Ethics approval was granted by the Walter Sisulu University Health Sciences Research Ethics & Biosafety Committee (WSU HREC 407/2025). Written informed consent was obtained from all participants or their legally authorized representatives prior to enrollment.

## Conflicts of Interest

The author declares no conflicts of interest.

## Use of Artificial Intelligence Tools

A large language model (CURIE, Springer) was used to assist with language editing and formatting of the manuscript. No AI tools were used for data collection, statistical analysis, or interpretation of results. All content was reviewed and approved by the author, who takes full responsibility for the work.

## Transparency Statement

The lead author, Luca D. Zambetti, affirms that this manuscript is an honest, accurate, and transparent account of the study being reported; that no important aspects of the study have been omitted; and that any discrepancies from the study as planned (and, if relevant, registered) have been explained.

## Supporting information

suppinfo LDZ Appendix A.

## Data Availability

The anonymised dataset supporting the findings of this study is available from the corresponding author.
