# Supplementary material for: Practical pH Testing for Nasogastric Tube Verification: A Prospective Method‐Comparison Study of Low‐Cost Handheld Meters and Colourimetric Strips
Source: Health Sci Rep. 2026 Mar 27;9(4):e72215. doi: 10.1002/hsr2.72215 (PMC13087631; doi:10.1002/hsr2.72215)
Supplement: Supplementary file 1 — suppinfo LDZ Appendix A. [file HSR2-9-e72215-s001.docx]

## **Appendix A**. Instrument Validation.

A bench validation was performed using standard buffer solutions (pH 4.00, 6.86, and 9.18) to assess basic accuracy under typical clinical use conditions.

The pH strips demonstrated accuracy within 0.5 pH units across all buffer solutions, consistent with their inherent 0.5-unit scale. The handheld pH meter showed close agreement with reference buffer values after rinsing with distilled water. When rinsed with tap water, the meter remained accurate but showed a slight downward shift at pH 4.00 and 6.86, likely due to residual ions or incomplete electrode cleaning. All deviations were small and within expected performance ranges for point-of-care devices (Table A1).

**Table A1.** Validation of the pH indicator strips and handheld pH meter using standard buffer solutions.

| **Buffer pH** | **4.00** | **6.86** | **9.18** |
| --- | --- | --- | --- |
| **pH indicator strip** | 4.0 | 6.5 | 9.0 |
| **pH meter (rinsed with distilled water)** | 3.98 | 6.85 | 9.17 |
| **pH meter (rinsed with tap water)** | 3.95 | 6.80 | 9.18 |
